# Pioneer Factor Improves CRISPR‐Based C‐To‐G and C‐To‐T Base Editing

**DOI:** 10.1002/advs.202202957

**Published:** 2022-07-21

**Authors:** Chao Yang, Xingxiao Dong, Zhenzhen Ma, Bo Li, Changhao Bi, Xueli Zhang

**Affiliations:** ^1^ Tianjin Institute of Industrial Biotechnology Chinese Academy of Sciences Tianjin 300308 China; ^2^ Key Laboratory of Systems Microbial Biotechnology Chinese Academy of Sciences Tianjin China; ^3^ School of Biological Engineering Dalian Polytechnic University Dalian 116034 China; ^4^ College of Life Sciences Nankai University Tianjin 300071 China

**Keywords:** CRISPR/Cas9, base editing, chromatin accessibility, pioneer factor

## Abstract

Base editing events in eukaryote require a compatible chromatin environment, but there is little research on how chromatin factors contribute to the editing efficiency or window. By engineering BEs (base editors) fused with various pioneer factors, the authors found that SOX2 substantially increased the editing efficiency for GBE and CBE. While SoxN‐GBE (SOX2‐NH3‐GBE) improved the editing efficiency at overall cytosines of the protospacer, SoxM‐GBE/CBE (SOX2‐Middle‐GBE/CBE) enabled the higher base editing at PAM‐proximal cytosines. By separating functional domains of SOX2, the SadN‐GBE (SOX2 activation domain‐NH3‐GBE) is constructed for higher editing efficiency and SadM‐CBE for broader editing window to date. With the DNase I assay, it is also proved the increased editing efficiency is most likely associated with the induction of chromatin accessibility by SAD. Finally, SadM‐CBE is employed to introduce a stop codon in the proto‐oncogene *MYC*, at a locus rarely edited by previous editors with high efficiency. In this work, a new class of pioneer‐BEs is constructed by fusion of pioneer factor or its functional domains, which exhibits higher editing efficiency or broader editing window in eukaryote.

## Graphical abstract

This work describes a novel destination for optimization of base editors with pioneer factors, especially SOX2 of which fusion to GEB and CBE enhances the editing outcomes via altering the chromatin accessibility. The N‐terminal fusion of SOX2 specially improves the GBE efficiency and fusion of it between APOBEC1 and Cas9 leads to a broader editing window for both editors.

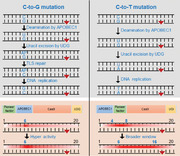

## 1 Introduction

BEs which combine a DNA deaminase with a catalytically impaired Cas nuclease can precisely manipulate targeted bases.^[^ ^1^ ^]^ To date, CBE (cytidine base editor),^[^ ^2^ ^]^ ABE (adenine base editor),^[^ ^3^ ^]^ and GBE (glycosylase base editor)^[^ ^4^ ^]^/CGBE (C‐to‐G base editors)^[^ ^5^, ^6^, ^7^ ^]^ were developed, catalyzing the conversion of C–G to T–A, A–T to G–C and C–G to G–C base pairs, respectively. BEs have enormous potential for applications in scientific research and clinical treatment of human genetic diseases.^[^ ^8^, ^9^, ^10^ ^]^ To improve the capacity of BEs, researchers have been actively developing novel systems with increased efficiency, improved specificity, and varied editing windows. Cheng et al. established CBEs with diversified editing windows via fusion to various cytidine deaminases,^[^ ^11^ ^]^ while Wang et al. fused APOBEC3A with nCas9 to efficiently perform base editing in methylated sequences.^[^ ^12^ ^]^ Wang et al. demonstrated that the specificity and efficiency could be enhanced by fusing more uracil glycosylase inhibitor units.^[^ ^13^ ^]^ It seems that most studies were focused on the optimization of BEs by protein engineering, while few investigations explored how the genomic environment affects the base editing process.

Nucleosome core particle consists of approximately 146 bp (base pairs) of DNA wrapped in 1.67 left‐handed superhelical turns around a histone octamer, which further organizes into higher‐order structures.^[^ ^14^ ^]^ As a consequence, DNA is sterically occluded, and in many cases occupied by chromatin regulators, which renders the DNA inaccessible to sequence‐specific DNA targeting proteins, such as transcriptional factors or Cas9 protein. It has been reported that heterochromatin states could hinder Cas9 access,^[^ ^15^, ^16^ ^]^ and it was also demonstrated that nucleosomes could partially block Cas9 binding to DNA in vivo.^[^ ^17^ ^]^ Correspondingly, the access of BEs to target loci could also be impeded by similar mechanisms. Additionally, the chromatin microenvironment was also reported to be associated with the DNA repair process,^[^ ^18^ ^]^ which might influence the C‐to‐G transition mediated via TLS (translesion DNA synthesis) repair.^[^ ^19^ ^]^ Thus, controlling and increasing the performance of BEs by manipulating DNA accessibility could be a direction for engineering a new class of BEs.

Various groups of proteins in eukaryotic cells were reported to impact DNA accessibility, such as chromatin remodelers, histone modifiers, pioneer factors, etc. Chromatin remodelers commonly facilitate DNA accessibility by interacting with histones.^[^ ^20^ ^]^ Histone modifiers were reported to induce an accessible chromatin environment by altering histone modifications^[^ ^21^, ^22^ ^]^ while pioneer factors are known to directly increase DNA accessibility through chromatin remodeling.^[^ ^23^ ^]^ Specifically, pioneer factors are considered to initiate chromatin opening and engage DNA sites for latter binding of transcriptional factors and similar sequence‐specific DNA targeting proteins.^[^ ^24^, ^25^ ^]^ For instance, pioneer factor FOXA1 (forkhead protein A1) was reported to open a compacted nucleosome to facilitate Androgen receptor (AR) binding to enhancers.^[^ ^26^ ^]^ Pioneer factor SOX2 (SRY‐box transcription factor 2) is known to initiate chromatin opening and facilitate transcriptional events.^[^ ^27^ ^]^ Pioneer factor PBX1 (pre‐B‐cell leukemia transcription factor 1) is thought to serve as a platform for MYOD (myogenic differentiation) binding in inactive chromatin,^[^ ^28^ ^]^ while pioneer factor PAX7 (paired box protein 7) is known to open targeted enhancers for the establishment and maintenance of cell identity.^[^ ^29^ ^]^ Despite it being reported that several chromatin remodelers were used to improve CRISPR‐Cas9 genome editing efficiency,^[^ ^30^ ^]^ their potential usage in base editing was largely undetermined. Furthermore, pioneer factors were known to exert the function of transcriptional activation^[^ ^27^ ^]^ that was also reported to potentially contribute to the Cas9‐dependent editing.^[^ ^31^ ^]^ Additionally, since the access of repair factors to DNA lesions requires an accessible chromatin environment, pioneer factors might positively affect the DNA repair process during base editing. Hence, we speculated that pioneer factors can be repurposed for the optimization of BEs.

In this study, we engineered BEs by fusing them with pioneer factors, which led to increased editing activity for GBE and CBE. Furthermore, we constructed the optimized SadN‐GBE and SadM‐CBE for higher editing efficiency and broader editing window, respectively. Finally, we demonstrated the potential of SadM‐CBE in silencing the proto‐oncogene *MYC* in eukaryocyte.

## 2 Results

### 2.1 Testing of Candidate Pioneer Factors for Optimization of BEs

**Figure 1:**
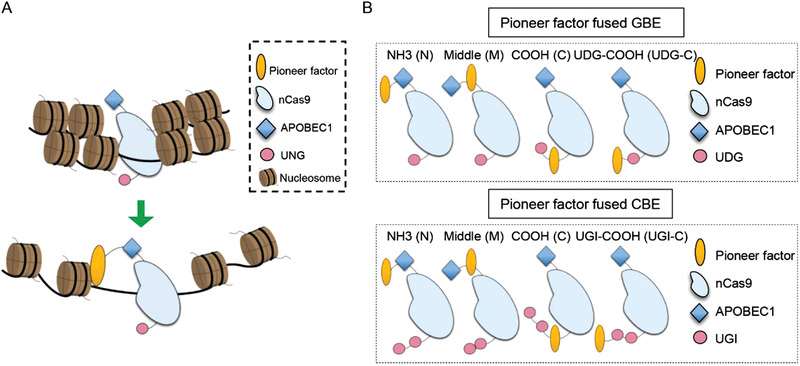
Schematic of pioneer factors fused base editors and its functional mechanism. A) Schematic of pioneer factors fusion to increase BE activity. B) Schematic of pioneer factors fusion strategy in CBE and GBE. APOBEC1 (apolipoprotein B mRNA editing enzyme catalytic subunit 1), nCas9 (nickase Cas9), UGI (uracil glycosylase inhibitor), UDG (uracil DNA glycosylase).

It has been demonstrated that Cas9 binding and cleavage are hindered by nucleosomes in eukaryotes.^[^ ^16^, ^17^ ^]^ A similar mechanism probably also restricts base editing events. Since pioneer factors are well known to open compacted chromatin, and endow the competence for transcriptional activation, we reasoned that fusion with pioneer factors might promote the editing activity of BEs (**Figure** **1**A). To test this hypothesis, four pioneer factors including FOXA1, SOX2, PBX1, and PAX7 were fused to GBE and CBE, respectively, to construct a series of pioneer‐BEs. Given that the relative orientation of Cas9 fusions might influence the editing activity of base editors, several groups of editor candidates were constructed with different arrangements of fused pioneer factors, deaminase, and Cas9 protein (Figure 1B). The constructed editors were expressed to edit genomic sites of mammalian cells, and the C‐to‐T or C‐to‐G conversion rates were determined by high‐throughput sequencing at four genomic sites.

**Figure 2:**
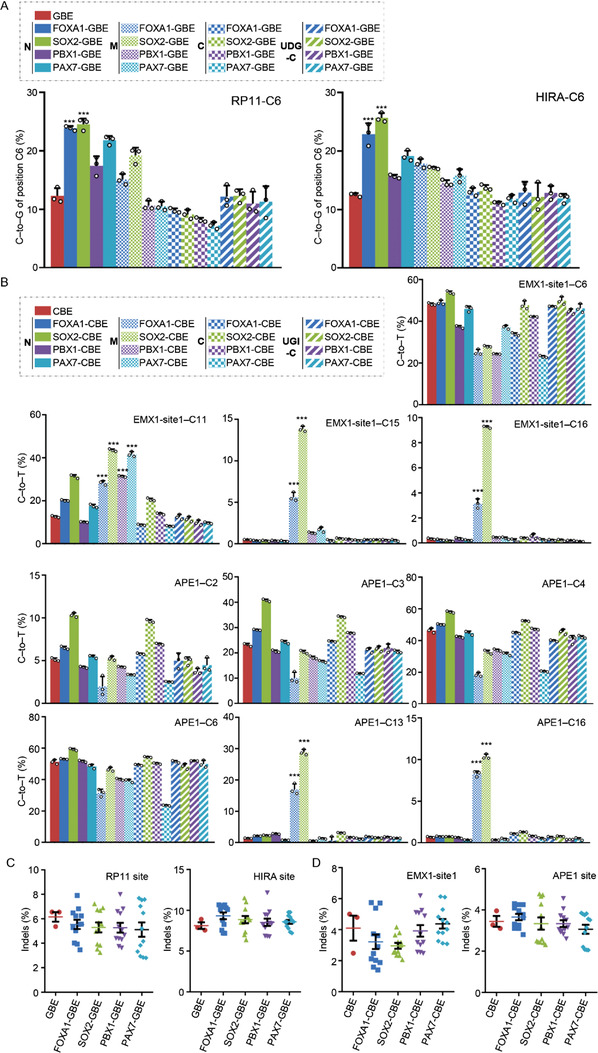
Testing of candidate pioneer factors for optimization of BEs. A) Base editing efficiency of GBE fused with variant pioneer factors in three arrangements at RP11 (left) and HIRA (right) loci in HEK293T cells. B) Base editing efficiency of CBE fused with variant pioneer factors in three arrangements at EMX‐site1 (upper) and APE1 (lower) loci in HEK293T cells. C) Comparison of the indel frequency across the protospacer of GBE and pioneer factors fused GBE at RP11 and HIRA loci in HEK293T cells. D) Comparison of the indel frequency across the protospacer of CBE and pioneer factors fused CBE at EMX‐site1 and APE1 loci in HEK293T cells. ***P < 0.001 (Student's *t*‐test).

Given that the editing center of GBE is primarily located at position C6 of the protospacer,^[^ ^4^ ^]^ its editing frequency in the GBE system was calculated. Our data revealed that the fusion of all tested pioneer factors at the amino‐terminal position of deaminase substantially enhances the editing activity of GBE by 22.34–105.25% (**Figure** **2**A). The results also indicated that the fusion of pioneer factors at the amino terminal and middle position in CBE (BE4max) had different editing outcomes. While fusion to amino terminal showed a slightly overall higher editing activity which is unlikely to be biologically relevant, that of carboxy terminal had a broader editing window, increasing from 2–11 to 2–16 (Figure 2B). Notably, GBE and CBE fused with the pioneer factor SOX2 were verified to be the most efficient among the pioneer‐BEs. Hence, the SOX2‐NH3‐GBE and SOX2‐Middle‐GBE/CBE were designated as SoxN‐GBE and SoxM‐CBE/CBE, respectively, and used for further analysis. Importantly, the indel frequency of the protospacer across these pioneer‐BEs remained at a low level at these four sites (Figures 2C,D). Further, to extend our research, we also constructed the SOX2 fused ABEmax for the analysis of A‐to‐G base editing. The results showed that SoxN‐ABE showed a substantially increased editing efficiency at several adenines of VISTA site and EMX1‐site3, but not for HEK4 site. However, we did not observe a higher editing efficiency at the PAM‐proximal adenines of SoxM‐ABE (Figure S1A,B, Supporting Information). Additionally, to demonstrate that the observed alterations were restricted to the function of the pioneer factor SOX2, we also constructed a transcriptional repressor ZNF704 fused GBE and CBE, of which function could inhibit chromatin accessibility via deacetylation.^[^ ^22^, ^32^ ^]^ Notably, the ZNF704 fused GBE in the amino position exhibited a decreased editing activity compared to the GBE (Figure S1C, Supporting Information). While the ZNF704 fused CBE in the middle position acted as a long linker between APOBEC1 and Cas9 and thus enabled PAM‐proximal editing, the overall editing efficiencies were significantly lower than SoxM‐CBE (Figure S1D, Supporting Information). Taken together, our results demonstrated that pioneer factors, especially SOX2, significantly increased the editing efficiency for GBE and CBE.

### 2.2 Fusion of SOX2 With GBE Increased Editing Activity

**Figure 3:**
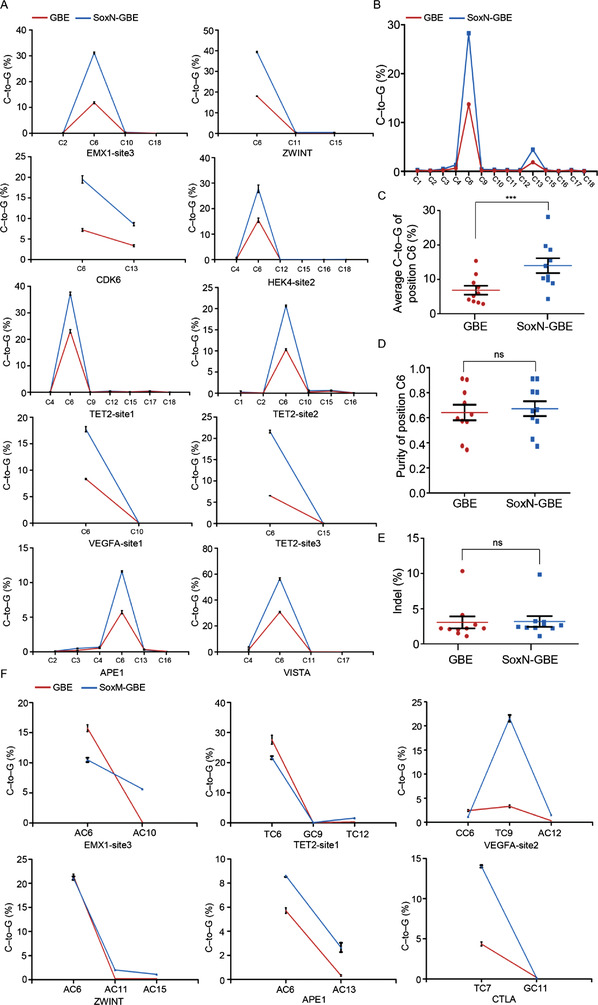
Fusion of SOX2 with GBE increased editing activity. A) Comparison of editing efficiency among GBE and SoxN‐GBE at ten endogenous genomic loci in HEK293T cells. B) Average C‐to‐G base editing efficiencies at C1‐C18 positions of protospacer from the ten targets of GBE and SoxN‐GBE. C) Average C‐to‐G base editing efficiencies at position C6 of the protospacer from the ten targets of GBE and SoxN‐GBE. D) Purity of C‐to‐G at position C6 of the protospacer from the ten targets of GBE and SoxN‐GBE. E) Comparison of the indel frequency across the protospacer of GBE and SoxN‐GBE at ten targets. F) Base editing efficiency of GBE and SadM‐GBE at six genomic sites in HEK293T cells. ns, not significant, ***P < 0.001 (Student's *t*‐test).

To further determine the effect of SOX2 for the optimization of GBE, editing experiments were performed at ten more genomic loci for SoxN‐GBE. The result showed that GBE and SoxN‐GBE both exhibited significant higher editing activity at position C6 of the protospacer, which was similar to the previous research^[^ ^4^ ^]^ (**Figures** **3**A,B). Notably, SoxN‐GBE was shown to have a higher editing activity compared to the control, with an average increase of 61.65‐231.13% at position C6 (Figures 3C). Importantly, SoxN‐GBE retained a similar indel rate and purity to that of the control at position C6 of the protospacer (Figure 3D,E). Further, considering that SoxM‐CBE might exhibit a higher editing of PAM‐proximal cytosines in our experiments, we suspected that this might also be the case for SoxM‐GBE. Six genomic sites with C7–C15 positions in different sequence contexts were edited using SoxM‐GBE. Our results showed that SoxM‐GBE exhibited a higher editing activity at PAM‐proximal cytosines than GBE (Figure 3F), even though the increase was only observed at loci containing Cs in an AC or TC context, but not in a GC context (Figure 3E). Specifically, GC9 in TET2‐site1 and GC11 in CTLA were not edited. Taken together, the data demonstrated that SoxN‐GBE and SoxM‐GBE exhibited a higher editing activity and broader editing window, respectively.

### 2.3 Fusion of SOX2 With CBE Enabled Higher Editing Activity at PAM‐Proximal Cytosines

**Figure 4:**
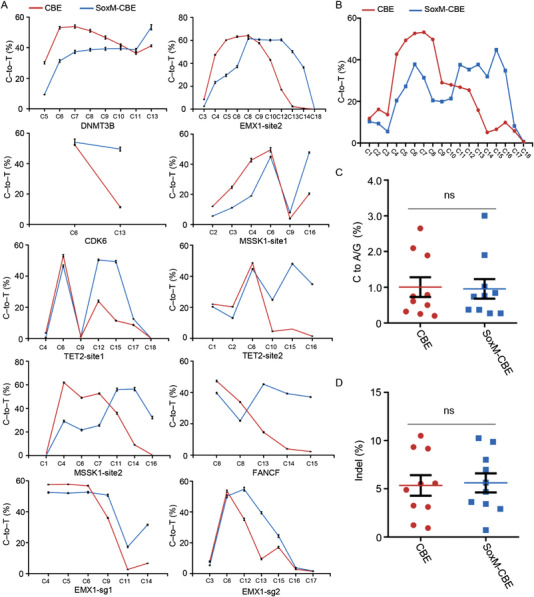
Fusion of SOX2 with CBE enabled higher editing activity at PAM‐proximal cytosines. A) Comparison of editing efficiency among CBE and SoxM‐CBE at ten endogenous genomic loci in HEK293T cells. B) Average C‐to‐G base editing efficiencies at C1‐C18 positions of protospacer from the ten targets of CBE and SoxM‐CBE. C) Frequency of C to A/G formation across the protospacer by CBE and SoxM‐CBE at ten targets. D) Comparison of the indel frequency across the protospacer of CBE and SoxM‐CBE at ten targets. ns, not significant.

Subsequently, we analyzed the effect of SOX2 for the optimization of CBE. The SoxM‐CBE was tested at ten genomic loci and our result showed that SoxM‐CBE exhibited a significantly improved editing efficiency at positions C10‐C18 with an increase of 11.31–580.95% relative to the control (**Figure** **4**A,B). The increase of editing efficiency was also effective in a GC context but with a lower increase than in the non‐GC context (GC9 in MSSK1‐site1). Importantly, the average frequency of indels and C to A/G byproducts remained similar to the control across the protospacer sequence (Figure 4C,D). Taken together, the data demonstrated that SOX2 fused CBE at the middle position exhibited a higher editing activity at PAM‐proximal cytosines.

### 2.4 Analysis of the Functional Domains of SOX2 Contributing to the Base Editing Performance

**Figure 5:**
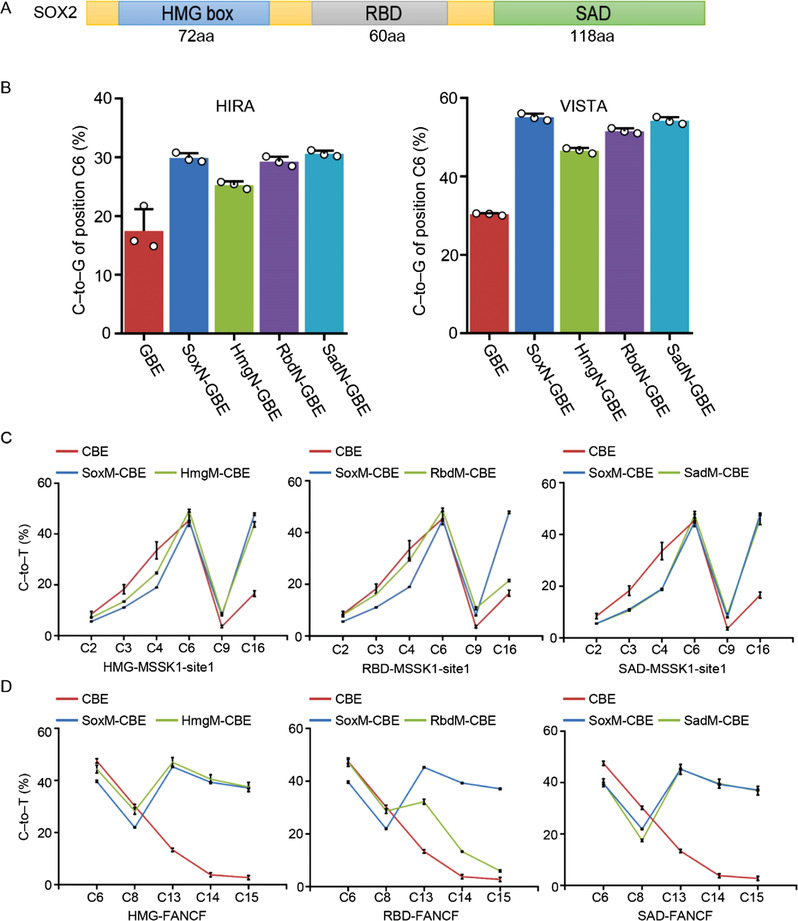
Analysis of the functional domains of SOX2 contributing to the base editing performance. A) Schematic of functional domains of pioneer factor SOX2. B) Base editing efficiency of GBE, SoxN‐GBE, and SOX2 domain fused GBEs at HIRA and VISTA site in HEK293T cells. C) Base editing efficiency of CBE, SoxM‐CBE, and SOX2 domain fused CBEs at MSSK1‐site1 site in HEK293T cells. D) Base editing efficiency of CBE, SoxM‐CBE, and SOX2 domain fused CBEs at FANCF site in HEK293T cells.

To obtain mechanistic insights into the increased editing activity of SoxM‐CBE and SoxN‐GBE, SOX2‐derived base editors were constructed using truncated functional domains of SOX2. It was reported that SOX2 is composed of three functional domains, including HMG (High mobility group), SAD (SOX2 activation domain), and a newly identified RBD (RNA binding domain) (**Figure** **5**A).^[^ ^27^, ^33^ ^]^ Hence, HmgN‐GBE (HMG‐NH3‐GBE), RbdN‐GBE (RBD‐NH3‐GBE), and SadN‐GBE (SAD‐NH3‐GBE) fusions were constructed for further investigation. Intriguingly, GBE constructs with HMG, SAD, and RBD at the amino‐terminal position all exhibited increased editing activity at position C6 of the protospacer, among which SadN‐GBE had the highest editing activity, which was nearly equal to that of SoxN‐GBE (Figure 5B). Furthermore, HmgM‐CBE (HMG‐Middle‐CBE), SadM‐CBE (SAD‐Middle‐CBE), and RbdM‐CBE (RBD‐Middle‐CBE) fusions were also constructed. The results showed that HMG/SAD at the middle position in CBE resulted in higher PAM‐proximal editing that was similar to SoxM‐CBE, but RBD not (Figure 5C,D). Given that the DNA binding function of HMG^[^ ^34^ ^]^ in base editors might induce other unexpected off‐target effects, SadM‐GBE and SadN‐CBE were employed for further application. In summary, our results proved that the functional domains of SOX2 had different effects on the efficacy of the base editing process.

### 2.5 Pioneer‐BEs Could Promote the Chromatin Accessibility at Target Genome Loci

**Figure 6:**
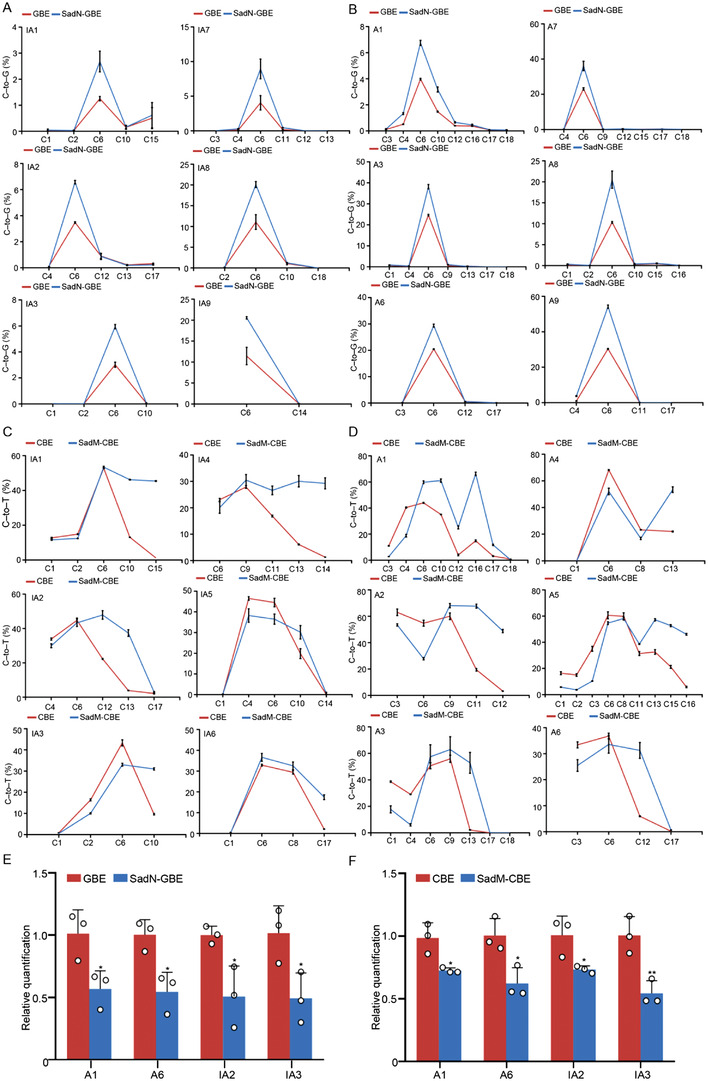
Pioneer‐BEs promote the chromatin accessibility at target genome loci. A) Comparison of editing efficiency between GBE and SadN‐GBE at six endogenous genomic loci from inaccessible chromatin in HEK293T cells. B) Comparison of editing efficiency between GBE and SadN‐GBE at six endogenous genomic loci from accessible chromatin in HEK293T cells. C) Comparison of editing efficiency among CBE and SadM‐CBE at six endogenous genomic loci from inaccessible chromatin in HEK293T cells. D) Comparison of editing efficiency among CBE and SadM‐CBE at six endogenous genomic loci from accessible chromatin in HEK293T cells. E) Comparison of chromatin state between GBE and SadN‐GBE at four loci in HEK293T cells. (F) Comparison of chromatin state among CBE and SadM‐CBE at four loci in HEK293T cells. *P < 0.05, **P < 0.01 (Student's *t*‐test); Accessible‐A, Inaccessible‐IA.

To explore the potential molecular mechanisms underlying the increased editing efficiency in SAD domain fused BEs, the protein expression and nuclear localization of BEs were compared between GBE and SadN‐GBE. The data hinted that there was no obvious alteration either in protein expression or nuclear localization between GBE and SadN‐GBE (Figure S2A, Supporting Information), suggesting that the SAD fusion might not affect the expression or nuclear localization of BEs. Notably, the transcriptional activation domains were reported to induce an open chromatin environment, thereby leading to chromatin decompaction.^[^ ^31^ ^]^ This encouraged us to test our pioneer‐BEs for base editing and the alteration of chromatin accessibility at differential chromatin regions. To address this issue, genomic loci located in differential chromatin environments (Accessible‐A; Inaccessible‐IA) were screened based on HEK293T DNase‐seq data, and were then edited using the pioneer‐BEs in HEK293T cells. Our data showed that SadN‐GBE was found to have higher editing activity across the protospacer, especially at C6, in both accessible and inaccessible chromatin regions (**Figure** **6**A,B). Additionally, SadM‐CBE exhibited a significantly increased editing efficiency at the PAM‐proximal cytosines compared to the CBE (Figure 6C,D).

Furthermore, to understand the effect of the pioneer factor in pioneer‐BEs on the editing of genomic sites from differential chromatin environments, the DNase I assay was performed to detect the alteration of chromatin states with pioneer‐BEs at four genomic sites. Our results showed that all pioneer‐BEs induced increased chromatin accessibility at the targeted loci compared to the control with no fused pioneer factor (Figure 6E,F). Taken together, these results indicated that pioneer‐BEs could promote chromatin accessibility, and their function in base editing was most likely associated with the induction of chromatin accessibility.

### 2.6 Investigation of Off‐Target Activity and Chromatin Remodeling by Pioneer‐BEs

**Figure 7:**
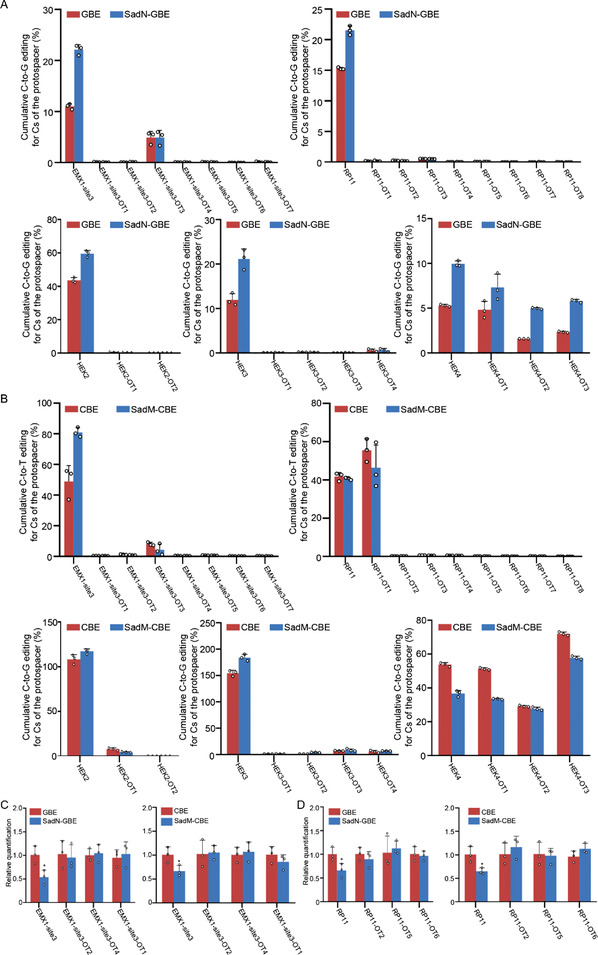
Investigation of off‐target activity and chromatin remodeling by pioneer‐BEs. A) Cumulative C‐to‐G editing for Cs of the protospacer between GBE and SadN‐GBE in HEK293T cells. B) Cumulative C‐to‐T editing for Cs of the protospacer among CBE and SadM‐CBE in HEK293T cells. C) Comparison of chromatin state of GBE, SadN‐GBE, CBE, and SadM‐CBE at EMX1‐site3 and its potential off‐targets in HEK293T cells. D) Comparison of chromatin state of GBE, SadN‐GBE, CBE, and SadM‐CBE at RP11 site and its potential off‐targets in HEK293T cells. *P < 0.05 (Student's *t*‐test).

Given that pioneer‐BEs are composed of canonical base editors and chromatin regulators, it is necessary to evaluate their potential off‐target effects. To figure out this issue, potential off‐target (OT) sites similar to each genomic site were screened using Cas‐OFFinder^[^ ^35^ ^]^ or based on the previously reported off‐target loci,^[^ ^36^, ^37^ ^]^ after which cumulative C‐to‐G or T editing frequencies were calculated for pioneer‐BEs and canonical BEs. Significantly, there was no evident increase in off‐target mutations induced by pioneer‐BEs (**Figure** **7**A,B). Furthermore, the alteration of chromatin states at potential off‐target sites was also analyzed due to the function of pioneer factors. Our data revealed that chromatin accessibility in these potential off‐target sites showed few alterations compared to the control (Figure 7C,D). Taken together, these results indicated that pioneer‐BEs exhibited low off‐target activity in terms of both base editing and change of chromatin states.

### 2.7 Characterization of Pioneer‐BEs in HeLa Cells

To further analyze the editing efficiency of pioneer‐BEs, we also tested them in HeLa cells, a cervical cancer cell line. Our data confirmed the higher editing activity of SadN‐GBE in HeLa cells at three genomic sites (Figure S3A,B,C). Additionally, the SadM‐CBE exhibited a similar increase of editing efficiency in HeLa cells across three genomic sites compared to the control (Figure S3D,E, Supporting Information). Similarly, the difference of indel rates across the protospacer between the control and pioneer‐BEs was not significant (Figure S3F, Supporting Information). Taken together, we demonstrated that pioneer‐BEs also exhibited increased editing efficiency in HeLa cells.

### 2.8 Highly Efficient Base Editing Using SadM‐CBE Potentially Induces Silencing of the Proto‐Oncogene MYC

**Figure 8:**
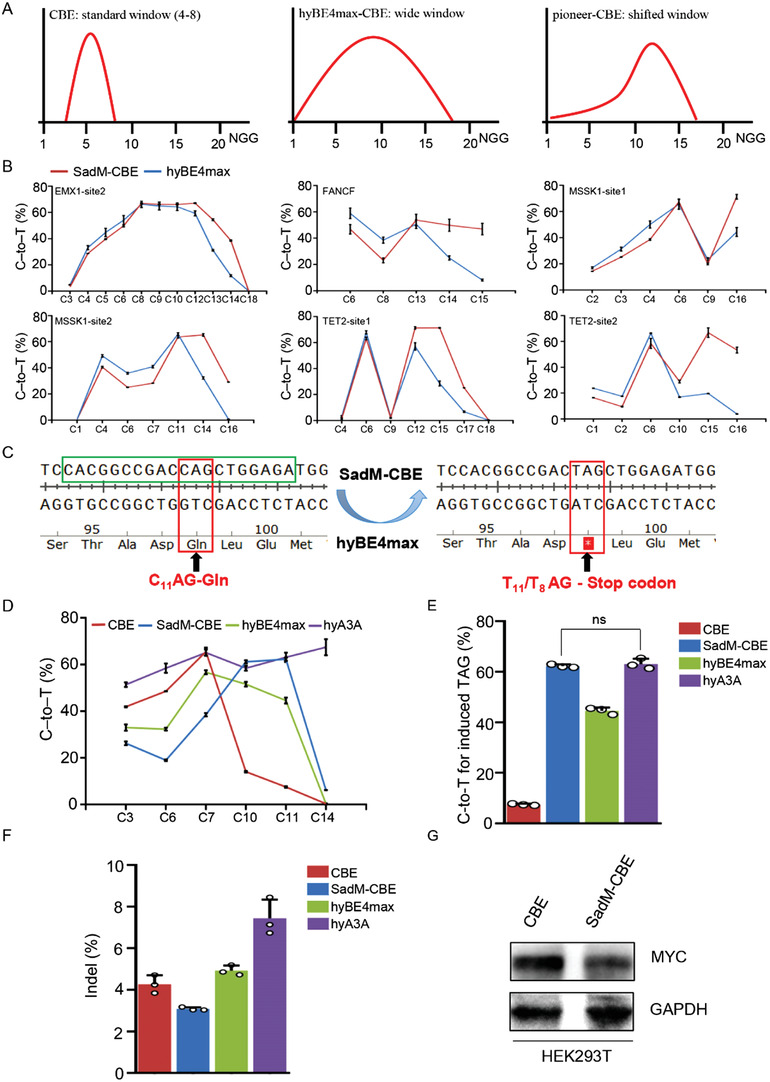
Highly efficient base editing using SadM‐CBE potentially induces silencing of the proto‐oncogene MYC. A) Schematic of editing window of canonical CBEs, wide‐window CBEs, and pioneer‐CBEs. B) Comparison of editing efficiency among SadM‐CBE and hyBE4max at ten endogenous genomic loci in HEK293T cells. C) Schematic of introducing stop codon via CBE. D) C‐to‐T editing of CBE, SadM‐CBE, hyBE4max, and hyA3A at MYC site in HEK293T cells. E) Comparison of C‐to‐T editing of CBE, SadM‐CBE, hyBE4max, and hyA3A at position C11 of MYC site. F) Comparison of indel frequency of CBE, SadM‐CBE, hyBE4max, and hyA3A at MYC site. G) Comparison of MYC protein level between CBE and SadM‐CBE by western blot. ns, not significant.

MYC is extensively recognized as a proto‐oncogene, and its amplification is frequently observed in malignant tumors.^[^ ^38^ ^]^ It has been reported that inhibition of this protein could result in the suppression of tumor growth.^[^ ^39^ ^]^ Given that SadM‐CBE had a higher efficiency in the PAM‐proximal regions (**Figure** **8**A), it was a promising tool to introduce a stop codon in MYC via base editing for oncogene disruption. To further verify the superiority of SadM‐CBE for the editing of PAM‐proximal cytosines, the hyBE4max including a DNA binding protein RAD51 between APOBEC1 and Cas9 that was reported to have a broader editing window^[^ ^40^ ^]^ was chosen for the comparison for the PAM‐proximal editing at six genomic loci. Our data indicated that SadM‐CBE showed a significantly higher PAM‐proximal editing of cytosines than hyBE4max at six genomic loci (Figure 8B). And then the potential encoding site of the MYC gene was screened and tested for the induction of stop codons using SadM‐CBE, hyBE4max, and hyA3A^[^ ^40^ ^]^ (Figure 8C). While the position C11 of the protospacer at the target loci was inefficiently edited by CBE, SadM‐CBE, hyBE4max, and hyA3A could effectively convert C into T at this locus with average efficiencies of 62.28%, 44.59%, and 63.11%, respectively (Figure 8D,E). Notably, the indel frequency of SadM‐CBE diminished compared to the other BEs (Figure 8F). Finally, to verify the silencing effect of the introduction of the stop codon, western blotting was performed with a specific anti‐MYC antibody. The data revealed that the protein level of MYC decreased in SadM‐CBE transfected cells compared to the control (Figure 8G). Taken together, our data demonstrated that SadM‐CBE could effectively introduce a stop codon in the proto‐oncogene MYC.

## 3 Discussion

Although it was reported that DNA binding proteins^[^ ^7^ ^]^ or DNA repair factors^[^ ^40^ ^]^ could promote higher base editing activity, to the best of our knowledge, there have been no researches about how chromatin factors influence the base editing outcomes. Here, by experimenting with several pioneer factors, we found that pioneer factor SOX2 fused GBE and CBE exhibited a significant alteration of base editing either at amino terminal or middle position. While the SoxN‐GBE showed an elevated editing activity, the SoxN‐CBE did not induce a biologically relevant increase of it. Although the pioneer factor might promote the access of Cas9 to the genomic sites, the editing effects of CBE and GBE were influenced by different molecular mechanisms, in which C‐to‐T conversion was mainly dependent on inhibition of UDG (uracil‐DNA glycosylase) activity^[^ ^2^ ^]^ and C‐to‐G conversion was recognized as a consequence of TLS via UDG and DNA polymerase in eukaryotes.^[^ ^19^, ^41^ ^]^ Accordingly, while the addition of more UGIs (Uracil glycosylase inhibitors) could result in a significantly increased editing efficiency and purity of CBE,^[^ ^13^ ^]^ the UDG activity might not be inhibited via improving chromatin accessibility. Conversely, the open chromatin environment might facilitate the assemble of TLS‐related polymerase or other repair factors to increase the C‐to‐G conversion. However, other functional mechanisms accounting for the increased editing efficiency could not be excluded. Further, in addition to the higher editing activity of SoxN‐GBE, the SoxM‐GBE and SoxM‐CBE constructs exhibited a higher PAM‐proximal editing. It is likely that SOX2 fusion at the middle position of BEs functions as a long linker sequence in addition to its pioneer activity. We hypothesized that the much longer linker and pioneer activity are both responsible for the better interaction between the deaminase and the R‐loop structure, thus enabling the higher PAM‐proximal editing. Nevertheless, we did not observe an increased PAM‐proximal editing in SoxM‐ABE, but a higher editing efficiency in SoxN‐ABE at several sites, which remains to be investigated in the future. Probably, these discrepancies might be explained by the differential working pattern of deaminase APOBEC1 and TadA or even the editing mechanism of CBE and ABE. More importantly, the indels and byproducts of the protospacer sequence across these base editors were found with similar frequencies to the control, demonstrating the safety of pioneer‐BEs. In general, we successfully constructed a new class of BEs fused with pioneer factor SOX2 which exhibited higher editing activity.

To further minimize the potential side effects of SoxM‐CBE and SoxN‐GBE, we then investigated the function of the distinct protein domains in SOX2. We found that while all truncated SOX2 domains fused GBE showed an increased editing activity, the HMG and SAD in the middle position of CBE were responsible for the broader editing window. It was reported that HMG domain of SOX2 could increase the chromatin accessibility via bending DNA^[^ ^27^ ^]^ and the SAD was recognized as a potential transactivation domain whose function was verified to increase the chromatin accessibility via recruiting histone acetyltransferase to alter binding capacity between histone and DNA.^[^ ^42^, ^43^ ^]^ Thus, both of them might promote the editing efficiency via altering chromatin accessibility. The RBD was reminiscent of a single‐stranded DNA binding domain as previously reported,^[^ ^40^ ^]^ which means that it might bind and stabilize the R‐loop structure for APOBEC1 interaction, thus contributing to the base editing process. Significantly, given the potential DNA binding property of HMG domain^[^ ^34^ ^]^ and relatively higher editing activity with SAD fusion, we recommend the usage of SadN‐CBE and SadM‐CBE for future application.

Next, considering the transcriptional activation might influence the chromatin accessibility,^[^ ^40^ ^]^ we wonder how the newly pioneer‐BEs worked in differential chromatin environments. Intriguingly, we observed that pioneer‐BEs increased the editing efficiency in both chromatin accessible and inaccessible regions. It was reported that nucleosomes are highly dynamic and frequently experience “site exposure” conformational fluctuations to orchestrate the access of DNA‐binding proteins.^[^ ^44^, ^45^, ^46^ ^]^ Accordingly, it is convincible that dynamic properties of the chromatin structure could transiently allow or prevent the access of base editors to the target DNA sequence, which is also corresponded with the previous conclusion that Cas9‐dependent editing was also improved with transactivation in accessible chromatin.^[^ ^40^ ^]^ Nevertheless, our results demonstrated the unique role of activation domain in contributing to the higher base editing activity and broader editing window. Above all, these findings imply that fusion with additional chromatin‐modulating partners could be a promising strategy to further optimize base editing. Theoretically, the function of pioneer factor domains in altering chromatin accessibility could also be applied in other genomic editing tools, but the effects might be variable and should be explored in detail for application.

Moreover, few increases in DNA off‐target effects and alterations of the chromatin state were identified with pioneer‐BEs at potential off‐target sites. Additionally, the increased editing efficiency of pioneer‐BEs was also reproduced in HeLa cells, which further demonstrated the application potential of pioneer‐BEs. Importantly, we also verified that SadM‐CBE exhibited a higher editing activity than hyBE4max in the PAM‐proximal region of the protospacer, which also confirmed the superiority of pioneer‐BEs. Finally, we also tested the application of SadM‐CBE in silencing of the proto‐oncogene MYC. We found that SadM‐CBE exhibited a similar editing efficiency but lower indel frequency than hyA3A at the targeted cytosine which could not be efficiently converted by CBE. Our data indicate that pioneer‐BEs could be an alternatively better choice for base editing of PAM‐proximal cytosines, especially for the silencing of MYC protein expression.

In summary, we exploited a new group of base editors fused with functional pioneer factors. These pioneer‐BEs were shown to have substantially increasing editing efficiency and a broader editing window. Our study further enriches the toolbox of base editing, and thus increases the application potential of BEs in genetic and non‐genetic therapies.

## 4 Experimental Section

### Cell Culture and Transfection

Cell lines used were obtained from the ATCC. HEK293T and HeLa cells were maintained in DMEM supplemented with 10% FBS in a humidified incubator equilibrated with 5% CO_2_ at 37°C. Cell lines used were no more than 20 passages. For transfection, cells were seeded in 24 well plates (Corning, USA) and carried out using polyethyienimine (Polysciences, USA) according to the manufacturer's instructions. 600 ng of BE plasmid and 300 ng of sgRNA‐expressing plasmid in total were transfected with 50 µl of Opti‐MEM (Gibco, USA) containing 2.7 µl of polyethyienimine. After 24 h transfection, fresh medium with 5 µg ml^−1^ puromycin (Merck, USA) was replaced. Cells were further cultured for 5 d for GBE and 3 d for CBE, and then genomic DNA was extracted via QuickExtract DNA Extraction Solution (Epicentre, USA). On‐target genomic regions of interest were amplified by PCR for high‐throughput DNA sequencing.

### Plasmid Construction

SOX2, PBX1, PAX7, and ZNF704 were amplified with Phusion DNA polymerase (NEB, USA) from HEK293T cDNA library. FOXA1 template was a gift from Prof. Shang Yongfeng. gRNA‐expression plasmids were assembled by the Golden Gate method with the protospacer sequence embedded in the primers, and *RNF2* sgRNA expression plasmids were used as the template.^[^ ^2^ ^]^ PCR products were gel purified, digested with DpnI restriction enzyme (NEB, USA), and assembled via Gibson assembly based on manufacturer's instructions. The main primers are listed in Table S1, Supporting information.

### Strains and Culture Conditions

*E. coli* DH5*α* was used as the cloning host and cultured at 37 °C in lysogeny broth (LB, 1% (w/v) tryptone, 0.5% (w/v) yeast extract, and 1% (w/v) NaCl). A 100 mg L^−1^ Ampicillin (Sigma. USA) was added to the medium for screen of positive cloning.

### High‐Throughput DNA Sequencing of Genomic DNA Samples and Data Analysis

The next‐generation sequencing library preparations were constructed following the manufacturer's protocol (VAHTS Universal DNA Library Prep Kit for Illumina). Briefly, purified PCR fragments were treated in one reaction with End Prep Enzyme Mix for end repair, 5’ phosphorylation, and dA tailing, which was followed by T‐A ligation to add adaptors to both ends. Each sample was then amplified with 4 cycles of PCR. Then the PCR products were purified using beads, validated using a Qsep100 (BiOptic, Taiwan, China), and quantified by a Qubit 3.0 Fluorometer (Invitrogen, Carlsbad, CA, USA).

Sequencing was carried out on Illumina HiSeq instrument according to the manufacturer's instructions (Illumina, San Diego, CA, USA). Briefly, a 2 × 150 paired‐end configuration was used. Image analysis and base calling were conducted by HiSeq Control Software (HCS) + RTA 2.7 (Illumina) on a HiSeq instrument. For pair‐end sequencing results, read 1 and read 2 were merged to generate a complete sequence according to their overlapping regions.

Amplicon sequencing data were analyzed with CRISPResso2 v.2.0.45 in batch mode,^[^ ^47^ ^]^ with window parameters set to ‐wc ‐10 ‐w 10. Briefly, the output file “Nucleotide_percentage_summary.txt” was analyzed for base conversion frequency, and “CRISPRessoBatch_quantification_of_editing_frequency.txt” was used to quantify the percentage of alleles that contain an insertion or deletion across the protospacer sequence for base editor experiments. And each point in position‐wise indel frequency is the sum of “Insertions_Left” and “Deletions” columns in the text file of “MODIFICATION_PERCENTAGE_SUMMARY.txt”. All clone oligos and deep sequencing oligos of sgRNA are listed in Table S2, Supporting information.

### DNase‐Seq Analysis

DNase‐seq data for HEK293T cells was obtained from the NCBI GEO (Gene Expression Omnibus) database. The DNase‐seq data (GEO: GSM1008573) were loaded into UCSC Genome Browser with GRCH38 (Genome Reference Consortium Homo sapiens 38) and the chromatin state of target sites was viewed.

### Detection of Chromatin Accessibility

Low‐input DNase I digestion assays were performed for the detection of chromatin accessibility as previously reported.^[^ ^48^ ^]^ Briefly, 6 × 10^5^ transfected HEK293T cells were resuspended in 60 µL lysis buffer and incubated on ice for 5 min. After that, DNase I (Sigma, USA) was added to the samples and further incubated at 37 °C for 5 min. Finally, the reaction was terminated with 60 µL stop buffer at 55°C for 1 h. The genomic DNA was extracted via the phenol‐chloroform method and analyzed by real‐time qPCR (SYBR GREEN, TOYOBO, Japan) with LightCycler 96 System. The genomic site of gapdh was used as the internal reference. The primers used are listed in Table S3, Supporting information.

### Western Blotting

The western blotting assay was performed as previously reported.^[^ ^32^ ^]^ Briefly, cellular extracts from HEK293T cells were prepared with lysis buffer (50 mM Tris‐HCl, pH8.0, 150 mM NaCl, 0.5% NP‐40) for 30 min at 4 °C and then denatured for 10 min at 95 °C. The cell lysates were resolved using 10% SDS‐PAGE gels and transferred onto acetate cellulose membranes. For incubation, membranes were incubated with Cas9 (Beyotime Biotechnology, China), MYC (Proteintech, USA), Histone H3 (ABclonal, USA) or GAPDH (ABclonal, USA) antibodies at 4 °C overnight followed by incubation with a secondary antibody (Proteintech, USA). Immunoreactive bands were visualized using western blotting luminal reagent (Millipore, USA) according to the manufacturer's recommendation.

### Statistics and Reproducibility

Unless otherwise noted, all data are presented as means ± S.D. from independent experiments. All statistical analyses were performed on at least three biologically independent experiments. The significance of the difference between the control and experiment group was calculated via student's *t*‐test using GraphPad Prism 8 (GraphPad Software). *P* < 0.05 was considered to be statistically significant.

## Conflict of Interest

C. Y., X. D., C. B., and X. Z. have submitted a patent application (application numbers 2021112817954) based on the results reported in this study.

## Supporting information

Supporting Information

## Data Availability

The data that support the findings of this study are openly available in NCBI Sequence Read Archive database at https://www.ncbi.nlm.nih.gov/sra/PRJNA765915, reference number 765915.
