# Supplementary material for: Pioneer Factor Improves CRISPR‐Based C‐To‐G and C‐To‐T Base Editing
Source: Adv Sci (Weinh). 2022 Jul 21;9(26):2202957. doi: 10.1002/advs.202202957 (PMC9475549; doi:10.1002/advs.202202957)
Supplement: Supplementary file 1 — Supporting Information [file ADVS-9-2202957-s001.pdf]

ADVANCED SCIENCE
Open Access

Supporting Information

for *Adv. Sci.*, DOI 10.1002/advs.202202957

Pioneer Factor Improves CRISPR-Based C-To-G and C-To-T Base Editing

Chao Yang, Xingxiao Dong, Zhenzhen Ma, Bo Li, Changhao Bi and Xueli Zhang**

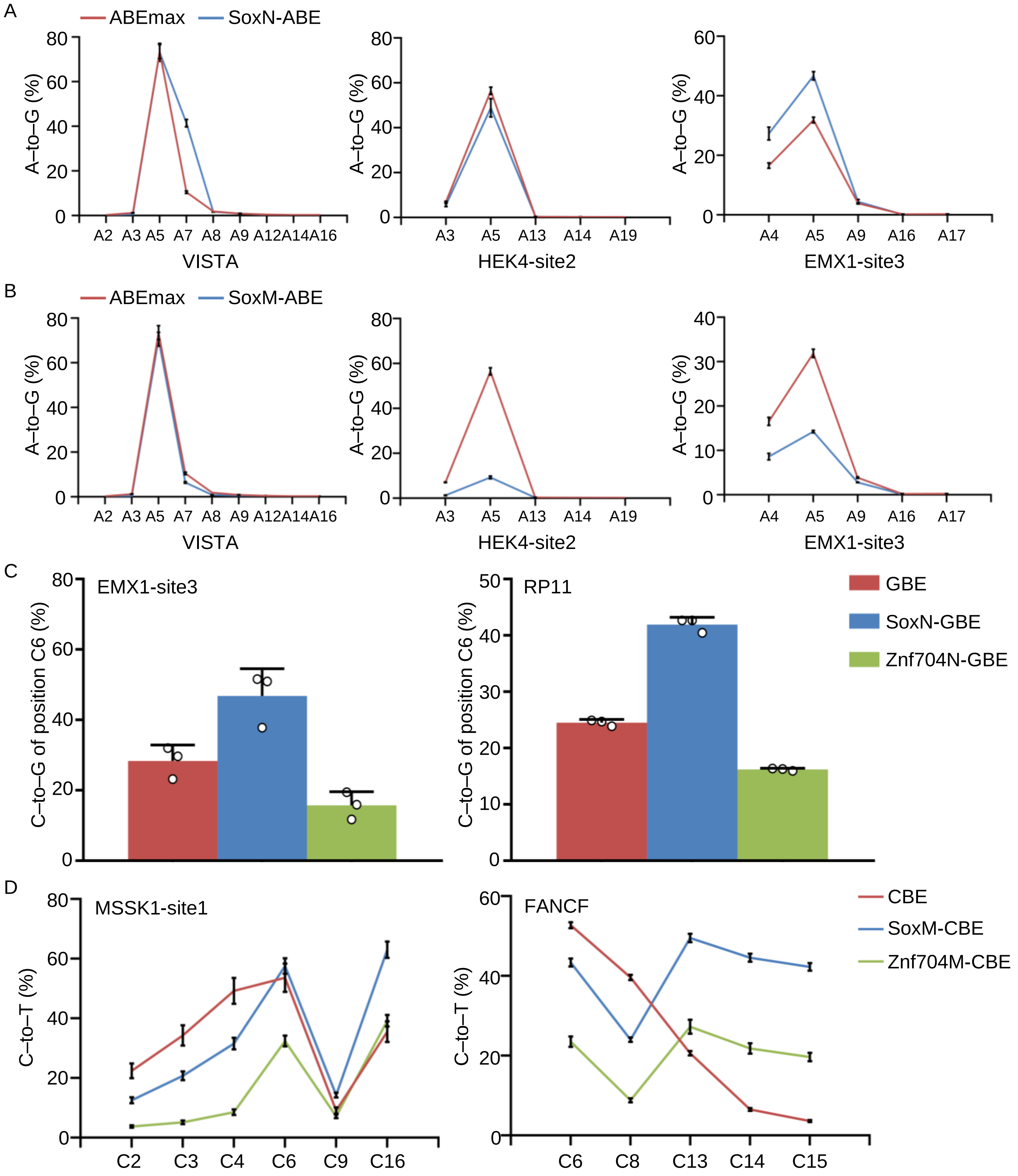

Figure S1. (A) Base editing efficiency of ABEmax and SoxN-ABE at VISTA, HEK4-site3 and EMX1-site3 in HEK293T cells. (B) Base editing efficiency of ABEmax and SoxM-ABE at VISTA, HEK4-site3 and EMX1-site3 in HEK293T cells. (C) Base editing efficiency of GBE, SoxN-GBE and Znf704N-GBE at EMX1-site3 and RP11 site in HEK293T cells. (D) Base editing efficiency of CBE, SoxM-CBE and Znf704M-CBE at MSSK1-site1and FANCF site in HEK293T cells.

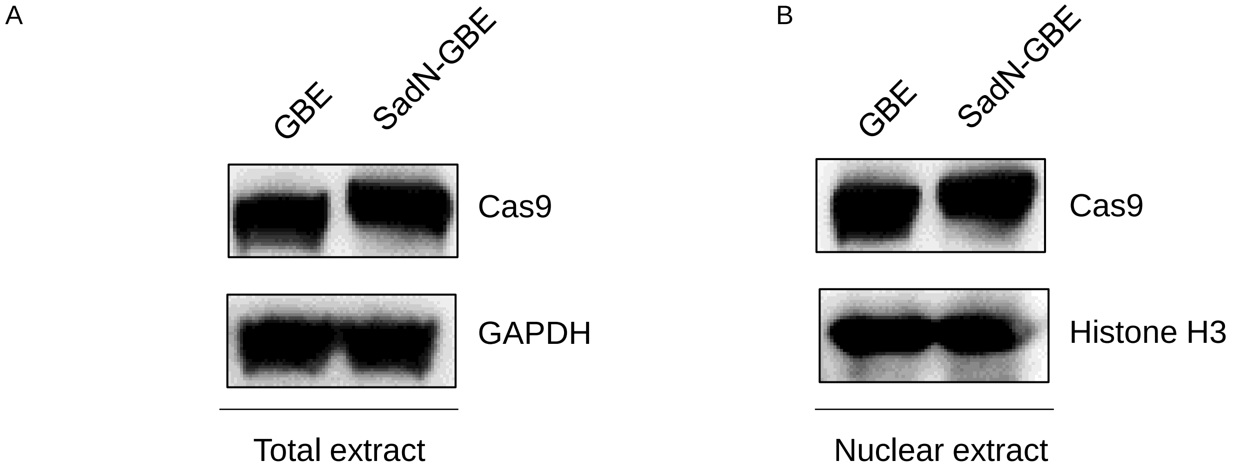

Figure S2. (A) Cas9 expression from HEK293T cell lysis between GBE and SadN-GBE. (B) Cas9 expression from HEK293T nuclear extract between GBE and SadN-GBE.

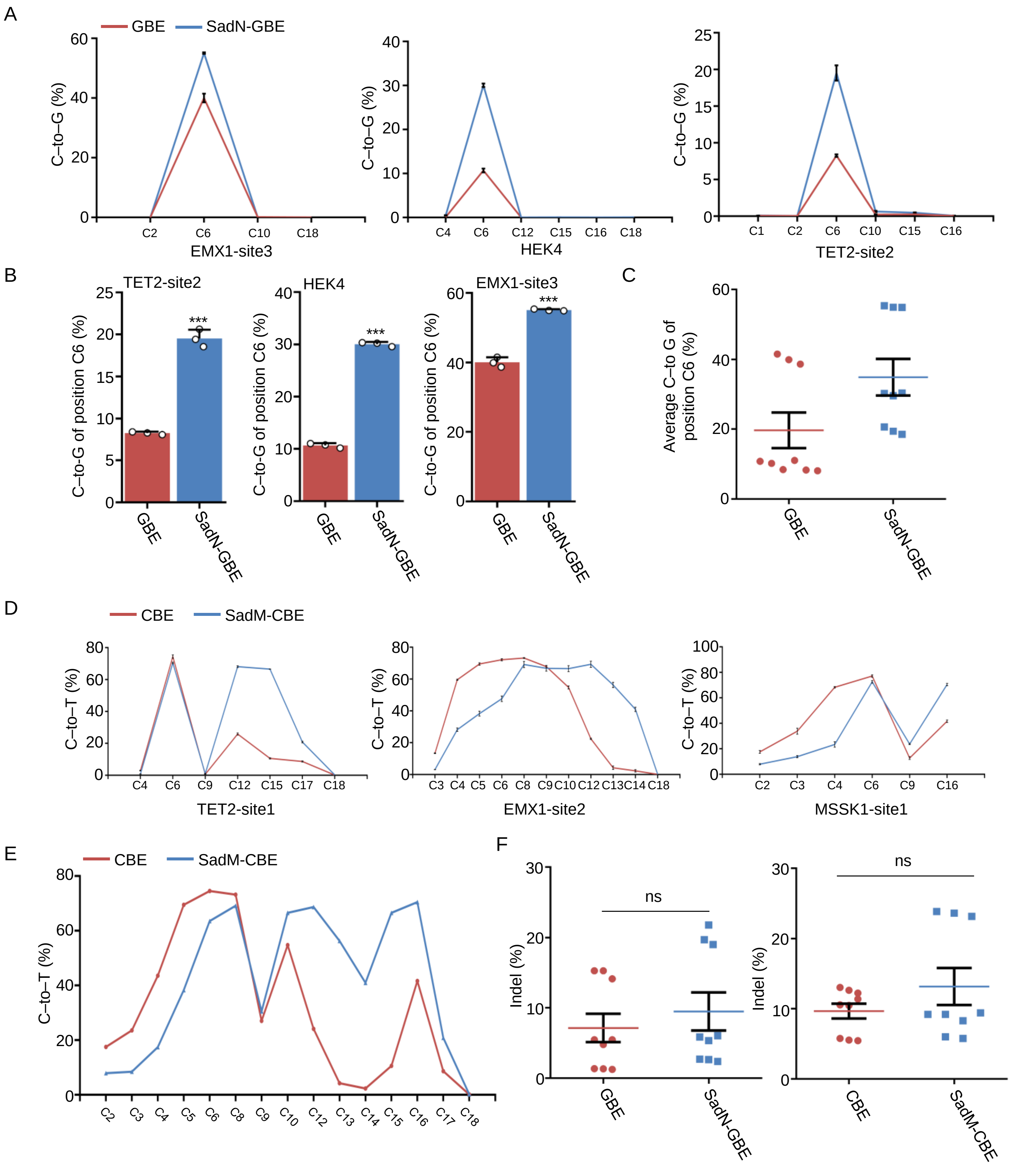

Figure S3. (A) Editing efficiency between GBE and SadN-GBE at three endogenous genomic loci in HeLa cells. (B) Efficiency at position C6 between GBE and SadN-GBE at three loci in HeLa cells. (C) Average C-to-G editing at position C6 at three targets edited by GBE and SadN-GBE in HeLa cells. (D) Efficiency among CBE and SadM-CBE at three endogenous genomic loci in HeLa cells. (E) Average editing efficiencies from three loci at C2-C18 of the protospacer by CBE and SadM-CBE. (F) The indel frequency across the protospacer of pioneer-BEs at three loci in HeLa cells. ***P<0.001 (Student's t-test); ns, not significant (Student's t-test).

Table S1: Clonging PCR oligos

Protein		Clone oligo (5'-3')
FOXA1	Oligo-up	ATGTTAGGAACTGTGAAGATGGAAGGGC
	Oligo-dn	GCTTCCTCCGCTACTTCCTCCGC
SOX2	Oligo-up	ATGTACAACATGATGGAGACGGAGC
	Oligo-dn	CATGTGTGAGAGGGGCAGTGTGC
PBX1	Oligo-up	ATGGACGAGCAGCCCAGGC
	Oligo-dn	GTTGGAGGTATCAGAGTGAACACTGCC
PAX7	Oligo-up	ATGGCGGCCCTTCCCGGC
	Oligo-dn	GGTGAACTGTTCCATCTGGCTGG
ZNF704	Oligo-up	ATGACCTTCACATTTCAGTC
	Oligo-dn	GTCGAGGAACCTCTGGCA
SOX2-HMG	Oligo-up	CGCGTCAAGCGGCCCATGA
	Oligo-dn	CCGGTATTTATAATCCGGGTGCTCCT
SOX2-RBD	Oligo-up	AAGAAGGATAAGTACACGCTGC
	Oligo-dn	GTAGCCCAGCTGGTC
SOX2-SAD	Oligo-up	GACGTGAGCGCCCTGCAGTACAACTCC
	Oligo-dn	CATGTGTGAGAGGGGCAGTGTGCC

Table S2-1: Clone oligos and deep sequencing olgios of sgRNA-Regular site

sgRNA	Target site sequence (5'-3')	Deep sequencing oligo (5'-3')	
APE1	CCCCACAGAAAACTACGGGC	Oligo-up	GCCTGGATTAAGAAGAAAGGATTAG
		Oligo-dn	GGAAGTGAAGGAACAGACATTTAG
EMX1-site1	GCTGGCATTACAAACCTGCC	Oligo-up	AGAGAGAGGCACAGATTTGGTGGG
		Oligo-dn	ATTATCAATTTCCACACTTTCAAC
RP11	GCAATCTGGGTGGAATGGTT	Oligo-up	GTCCAGTGAGTATTGTCTAAGG
		Oligo-dn	GAGGTCTCCAATTGTCTACC
HIRA	GTATTCTAGAATGCAGGGCA	Oligo-up	GTGTGGAAGATAGATGAG
		Oligo-dn	CTGCCATCACAATTCTAGTC
DNMT3B	AGAGCCCCCCCTCAAAGAGA	Oligo-up	GGCTGTTTGTCTTGTGGC
		Oligo-dn	AAGGCAGCTGGGATGAGTG
EMX1-site2	TGCCCCTCCCTCCCTGGCCC	Oligo-up	TGAGTGTTGAGGCCCCAGT
		Oligo-dn	CTGCTTCGTGGCAATGCG
CDK6	GTGAACAATTATCAAAATGG	Oligo-up	AGACTTTAAGCCAGAGACAC
		Oligo-dn	CTAGCCATGATGTGCTGC
MSSK1-site1	GACCCACGGCGGGGATCAGGG	Oligo-up	GTGTTCAATGGGCGGTACCACG
		Oligo-dn	CTTGCGCCTGCAATACCGAGAC
TET2-site1	TTGCTCTGCTTCTTCTCCCA	Oligo-up	ATCCCAACCTCCCACCTCG
		Oligo-dn	AGTGGAGACCTGGGCTGC
TET2-site2	CCTTTCTAACAAATCCTAAA	Oligo-up	GCTGCTTTCGTAGAGAAGC
		Oligo-dn	GACACTACAGGGTTTGCT
MSSK1-site2	CGTCGCCGATCTTCACAGGG	Oligo-up	TGGGCCAGATGGTCAATGG
		Oligo-dn	AGCCAGACGGTGGAGAAGT
FANCF	AAGTTCGCTAATCCCGGAAC	Oligo-up	GGCTGCACAACCAGTGGA
		Oligo-dn	CCAGGCTCTCTTGGAGTGTC
EMX-sg1	GCTCCCATCACATCAACCGG	Oligo-up	GTGAAGGTGTGGTTCCAGAA
		Oligo-dn	CTCGTGGGTTTGTGGTTG
EMX-sg2	GACATCGATGTCCTCCCCAT	Oligo-up	GTGAAGGTGTGGTTCCAGAA
		Oligo-dn	CTCGTGGGTTTGTGGTTG
TET2-site3	AGATTCTGAATGAGCAGGAG	Oligo-up	TCGGGGTAAGCCAAGAAAG
		Oligo-dn	CTTGTCATGGTAATTAGCACTT
ZWINT	TGAAACAAAACATACAATAG	Oligo-up	CGAAAACAGAAAGTGGGAAAGAT
		Oligo-dn	ACAGTGAGCTCATCTGACT
HEK4-site2	GTACACTTGTGCAACCTCAC	Oligo-up	ATTTCTGTCAACCCACTACAGC
		Oligo-dn	GGCACAAGAATAATAGACCCTGG
VEGFA-site1	ATGTTCTTGCTGTTGTTGTT	Oligo-up	CAGTGGCTCACACCTAGT
		Oligo-dn	CGTGAAATCAATTGTTTAGAGAGGA
VISTA	GAACACAAAGCATAGACTGC	Oligo-up	GGCTGAGCTAACTGTGACAGCATGTG
		Oligo-dn	CCCAAGTGAGAAGCCAGTGGAATAC
VEGFA-site2	GACCCCCTCCACCCCGCCTC	Oligo-up	TTGGGATCCCGCAGCTGA
		Oligo-dn	TCCGAAGCGAGAACAGCC
CTLA	CTCCCTCAAGCAGGCCCCGC	Oligo-up	TTCCAGGGCAGCAGAAGAA
		Oligo-dn	GCAGATGTAGTGTTTCCACAGG
EMX1-site3	GCTAACTTACTGTGTAACCC	Oligo-up	AGATGGTTTGGACAGAGCTTCC
		Oligo-dn	TACCTTAAAGGGAAGGGACTAGGG
MYC-site	CACGGCCGACCAGCTGGAGA	Oligo-up	CCCTCCTACGTTGCGGTCA
		Oligo-dn	CGAGAAGCCGCTCCACAT

Table S2-2: Clone oligos and deep sequencing olgios of sgRNA-Chromatin environment

Name	sgRNA	Target site sequence (5'-3')		Deep sequencing oligo (5'-3')
A1	RIOK2	GGCCACATTCACTTTCCCCA	Oligo-up	CGGCGGGTTTCTTACCGC
			Oligo-dn	TATACTAAGACGCAGCGCGTGC
A2	CAST	ATCTACTTCTCCGGATTGTT	Oligo-up	CACACACACACAGACCAATTTGTTTTTG
			Oligo-dn	AGTTGTGCAACGAGAGAGCG
A3	AFF1	CTGCACTTCAGACGAGCCCG	Oligo-up	TTGTGCTGTTGCTGGGCAGG
			Oligo-dn	AATCCTGATGGACATCCGGGC
A4	MED28	CAGTTCACGTTTCAGAAAAA	Oligo-up	TTCAGGCAGCTCCAGGCGC
			Oligo-dn	CCCATTTGTGAATTAGTTGCTCGGATCCC
A5	SLC11A1	CCCATCTCTTCTCTCCTTGA	Oligo-up	TTCCTGGGGACCCAGCAGG
			Oligo-dn	TTGCCCAGGTACCCTCTGCA
A6	RNF2	GTCATCTTAGTCATTACCTG	Oligo-up	ACGGAACTCAACCATTAAG
			Oligo-dn	AGTGTTAGCCAACATACAG
A7	TET2-site1	TTGCTCTGCTTCTTCTCCCA	Oligo-up	ATCCCAACCTCCCACCTCG
			Oligo-dn	AGTGGAGACCTGGGCTGC
A8	TET2-site2	CCTTTCTAACAAATCCTAAA	Oligo-up	GCTGCTTTCGTAGAGAAGC
			Oligo-dn	GACACTACAGGGTTTGCT
A9	VISTA	GAACACAAAGCATAGACTGC	Oligo-up	GGCTGAGCTAACTGTGACAGCATGTG
			Oligo-dn	CCCAAGTGAGAAGCCAGTGGAATAC
IA1	AC110614.1	CCTTACAGACTAGAACAAAG	Oligo-up	CTTACTGGGCAGTTTTTATTGTCTTTCAACAC
			Oligo-dn	GACAAAAGTGTCTAGTCACCTATAAGAAAAATCCC
IA2	AK123816-I1	GAACTCTTATTCCTTGCTAG	Oligo-up	TGAAGAAAGCTGTCCTAAACATCCGT
			Oligo-dn	TACTGTAAAAGATAACAATACCAAGTGCTGG
IA3	AK123816-2-I11	CCATTCTAACTGGTGTGAGA	Oligo-up	CTTCAAGAACCATGTTATCATTTTCCGCA
			Oligo-dn	TCGCCACACTGACTTCCCA
IA4	chr5-q21.1	TTTAACTTCTCTCCTGAGTA	Oligo-up	TCCATTGTTCAACTCATTCTGCAATTAAGC
			Oligo-dn	AGTCATAATCTAAATTGTATTTCCTGGCAGTAAGT
IA5	HCN1-site1	CTTCACTGTCTTGCTAAATA	Oligo-up	TCTGCTTGTTCATATTGGCAGTAACAAGGT
			Oligo-dn	TATATTAGGAAACTACAAATTGTAACCTCTTATCCCTGA
IA6	ch2-p12	CTTTTCTCTGTAATATCACG	Oligo-up	ACGGAACTCAACCATTAAG
			Oligo-dn	TAGTGCCTTTATAAAAGAGGCTCCAGAGA
IA7	HCN1-site2	AACCTCTTATCCCTGATGGT	Oligo-up	TAAGATGCAGGACACAGTGTGCTAC
			Oligo-dn	GGTTAAATGAGGTTCCTTGTCTGTCTCT
IA8	EMX1-site3	GCTAACTTACTGTGTAACCC	Oligo-up	AGATGGTTTGGACAGAGCTTCC
			Oligo-dn	CTACCTTAAAGGGAAGGGACTAGGG
IA9	HIRA	GTATTCTAGAATGCAGGGCA	Oligo-up	GTGTGGAAGATAGATGAG
			Oligo-dn	CTGCCATCACAATTCTAGTC

Table S2-3: Clone oligos and deep sequencing olgios of sgRNA- Off-target site

sgRNA	Target site sequence (5'-3')		Deep sequencing oligo (5'-3')
EMX1-site3-OT1	GCTAACTTGCTGTGTGACCC	Oligo-up	TATGGTGGTGGTTGAATCAATCTATATTATGCTA
		Oligo-dn	ACATGTATTCTGCTCTTCTGTCCTCAC
EMX1-site3-OT2	CCTAACTTACTGTGTAAGCC	Oligo-up	CTGCAGGAGAAATAATCAGAGTTATAAAGTATATACCT
		Oligo-dn	GAAGGTCAAGGGATAGAAGGTTGCA
EMX1-site3-OT3	GCAATCTTACTCTGTCACCC	Oligo-up	CCTTGAGCCCAGGAGTAAGAGG
		Oligo-dn	GCATGGCCTTTGCCCTGAT
EMX1-site3-OT4	GCTTACTCACTGTGTATCCC	Oligo-up	GGGAGTTAGGAATTTGGGTTCTGAAGG
		Oligo-dn	GAGGCTTCATCTTAAATGGTTGTCAGGT
EMX1-site3-OT5	GCTGCCTTGCTGTGTGACCC	Oligo-up	ATGGAAGGAGCATTGGACAGAGAG
		Oligo-dn	GAGAATAATAGGCTGTGGCTAGGGTATTT
EMX1-site3-OT6	GCTATTTAACTGTGTGACCC	Oligo-up	CTCTATGGTGCATGGTTTGAGGATATTATCTT
		Oligo-dn	GCTCAAAGGGATCTCAGGTTTCTTATCAC
EMX1-site3-OT7	GGGTCACACAGCAAGTTAG	Oligo-up	AGGAAAGTCCACAGAACACAGAAATAGTTAAAG
		Oligo-dn	TGCCATCATCAGACAACACTTTACAAAATG
RP11-OT1	GGAAGCTGGGTGACATGGTT	Oligo-up	GTGGGAATTATGGGAGCTACAATTCAAGA
		Oligo-dn	ACTTTGTATTGGTCGCAAAGAAGGAAC
RP11-OT2	GCAATCGGGCTGGAATCTTT	Oligo-up	AGAGATGGCCTCCCAGATGTC
		Oligo-dn	GGCTTGTAGGAACGCTCAGTGA
RP11-OT3	GCAGTCACTGTGGAATGGTT	Oligo-up	TGCAAGCCCAAACAACAATATACCATTTC
		Oligo-dn	CCTGTGTTTAGCTTCTTATATCGCAGTTCT
RP11-OT4	GCTAACTGGGTGGTATGGTT	Oligo-up	CCCGTGAGTTAATGGTCTCCCTC
		Oligo-dn	CACCATAGTTGGCTCCTCTGGG
RP11-OT5	GCAACCTGGGTGGACTGTTT	Oligo-up	AGGGACAAGAGGATTGAGGTCAC
		Oligo-dn	GGAGTTTGGTGCCTGATACCATTG
RP11-OT6	GCAATCTGGGGTATATGGTT	Oligo-up	AGCACAACTCTGCAAGCTACTC
		Oligo-dn	ACAGCTTACACGGACTTCTCCTTT
RP11-OT7	GCAATCGGGATGGAATTGTG	Oligo-up	TGGCAGGCAGATCACAAACAG
		Oligo-dn	CCTCAGTGACAAGTGCATACCTCC
RP11-OT8	GCAATTTGTGTGGAATGGTT	Oligo-up	TCTGAACTCTTTGCACTTTGGAGTGAC
		Oligo-dn	CAAATACCCTGTGGTTTGGCCC
HEK2	GAACACAAAGCATAGACTGC	Oligo-up	GGCTGAGCTAACTGTGACAGCATGTG
		Oligo-dn	CCCAAGTGAGAAGCCAGTGGAATAC
HEK2-OT1	GAACACAATGCATAGATTGC	Oligo-up	GTGTGGAGAGTGAGTAAGCCA
		Oligo-dn	ACGGTAGGATGATTTCAGGCA
HEK2-OT2	AAACATAAAGCATAGACTGC	Oligo-up	CACAAAGCAGTGTAGCTCAGG
		Oligo-dn	TTTTTGGTACTCGAGTGTTATTCAG
HEK3	GGCCCAGACTGAGCACGTGA	Oligo-up	GGAAACGCCCATGCAATTAGTC
		Oligo-dn	CTTGTCAACCAGTATCCCGGTG
HEK3-OT1	CACCCAGACTGAGCACGTGC	Oligo-up	TCCCCTGTTGACCTGGAGAA
		Oligo-dn	CACTGTACTTGCCCTGACCA
HEK3-OT2	GACACAGACTGGGCACGTGA	Oligo-up	TTGGTGTTGACAGGGAGCAA
		Oligo-dn	CTGAGATGTGGCAGAAGGG
HEK3-OT3	AGCTCAGACTGAGCAAGTGA	Oligo-up	TGAGAGGGAACAGAAGGGCT
		Oligo-dn	GTCCAAAGGCCCAAGAACCT
HEK3-OT4	AGACCAGACTGAGCAAGAGA	Oligo-up	CCTAGCACTTTGGAAGGTCG
		Oligo-dn	GCTCATCTTAATCTGCTCAGCC
HEK4	GGCACTGCGGCTGGAGGTGG	Oligo-up	AAGGAAGGGAGGAAGGGC
		Oligo-dn	ACACACACACAGGCCTG
HEK4-OT1	TGCACTGCGGCCGGAGGAGG	Oligo-up	GGCATGGCTTCTGAGACTCA
		Oligo-dn	CCCTTGCACTCCCTGTCTTT
HEK4-OT2	GGCTCTGCGGCTGGAGGGGG	Oligo-up	TTTGGCAATGGAGGCATTGG
		Oligo-dn	GAAGAGGCTGCCCATGAGAG
HEK4-OT3	GGCATCACGGCTGGAGGTGG	Oligo-up	TTCCACCAGAACTCAGCCC
		Oligo-dn	CCTCGGTTCCTCCACAACAC

Supplementary Table S3: PCR oligo of genomic sites from different chromatin environments

Gene		Clone oligo (5'-3')
A1	Oligo-up	CGGCGGGTTTCTTACCGC
	Oligo-dn	TATACTAAGACGCAGCGCGTGC
A6	Oligo-up	ACGGAACTCAACCATTAAG
	Oligo-dn	AGTGTTAGCCAACATACAG
IA2	Oligo-up	TGAAGAAAGCTGTCCTAAACATCCGT
	Oligo-dn	TACTGTAAAAGATAACAATACCAAGTGCTGG
IA3	Oligo-up	CTTCAAGAACCATGTTATCATTTTCCGCA
	Oligo-dn	TCGCCACACTGACTTCCCA
EMX1-site3	Oligo-up	AGATGGTTTGGACAGAGCTTCC
	Oligo-dn	CTACCTTAAAGGGAAGGGACTAGGG
RP11	Oligo-up	GTCCAGTGAGTATTGTCTAAGG
	Oligo-dn	GAGGTCTCCAATTGTCTACC
EMX1-site3-OT1	Oligo-up	TATGGTGGTGGTTGAATCAATCTATATTATGCTA
	Oligo-dn	ACATGTATTCTGCTCTTCTGTCCTCAC
EMX1-site3-OT2	Oligo-up	CTGCAGGAGAAATAATCAGAGTTATAAAGTATATACCT
	Oligo-dn	GAAGGTCAAGGGATAGAAGGTTGCA
EMX1-site3-OT4	Oligo-up	CTCTATGGTGCATGGTTTGAGGATATTATCTT
	Oligo-dn	GCTCAAAGGGATCTCAGGTTTCTTATCAC
RP11-OT2	Oligo-up	AGAGATGGCCTCCCAGATGTC
	Oligo-dn	GGCTTGTAGGAACGCTCAGTGA
RP11-OT5	Oligo-up	AGGGACAAGAGGATTGAGGTCAC
	Oligo-dn	GGAGTTTGGTGCCTGATACCATTG
RP11-OT6	Oligo-up	AGCACAACTCTGCAAGCTACTC
	Oligo-dn	ACAGCTTACACGGACTTCTCCTTT
GAPDH	Oligo-up	TCTGGTGGCTGGCTCAGAA
	Oligo-dn	TGGTGGTCCAGGGGTCTTAC
